# Supplementary material for: Regulation of presynaptic Ca2+ channel abundance at active zones through a balance of delivery and turnover
Source: eLife. 2022 Jul 14;11:e78648. doi: 10.7554/eLife.78648 (PMC9352347; doi:10.7554/eLife.78648)
Supplement: Figure 3—source data 2. [file elife-78648-fig3-data2.pdf]

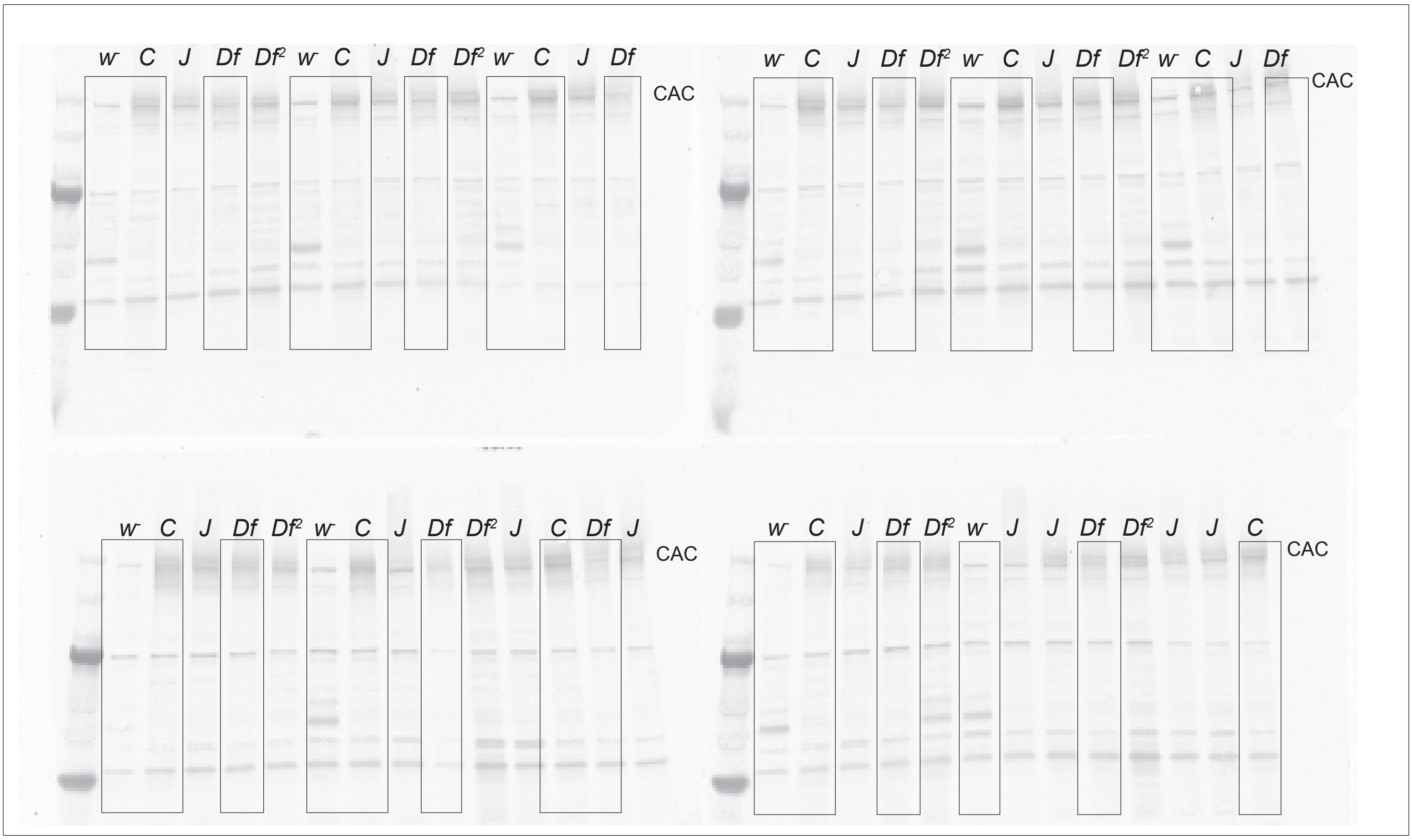

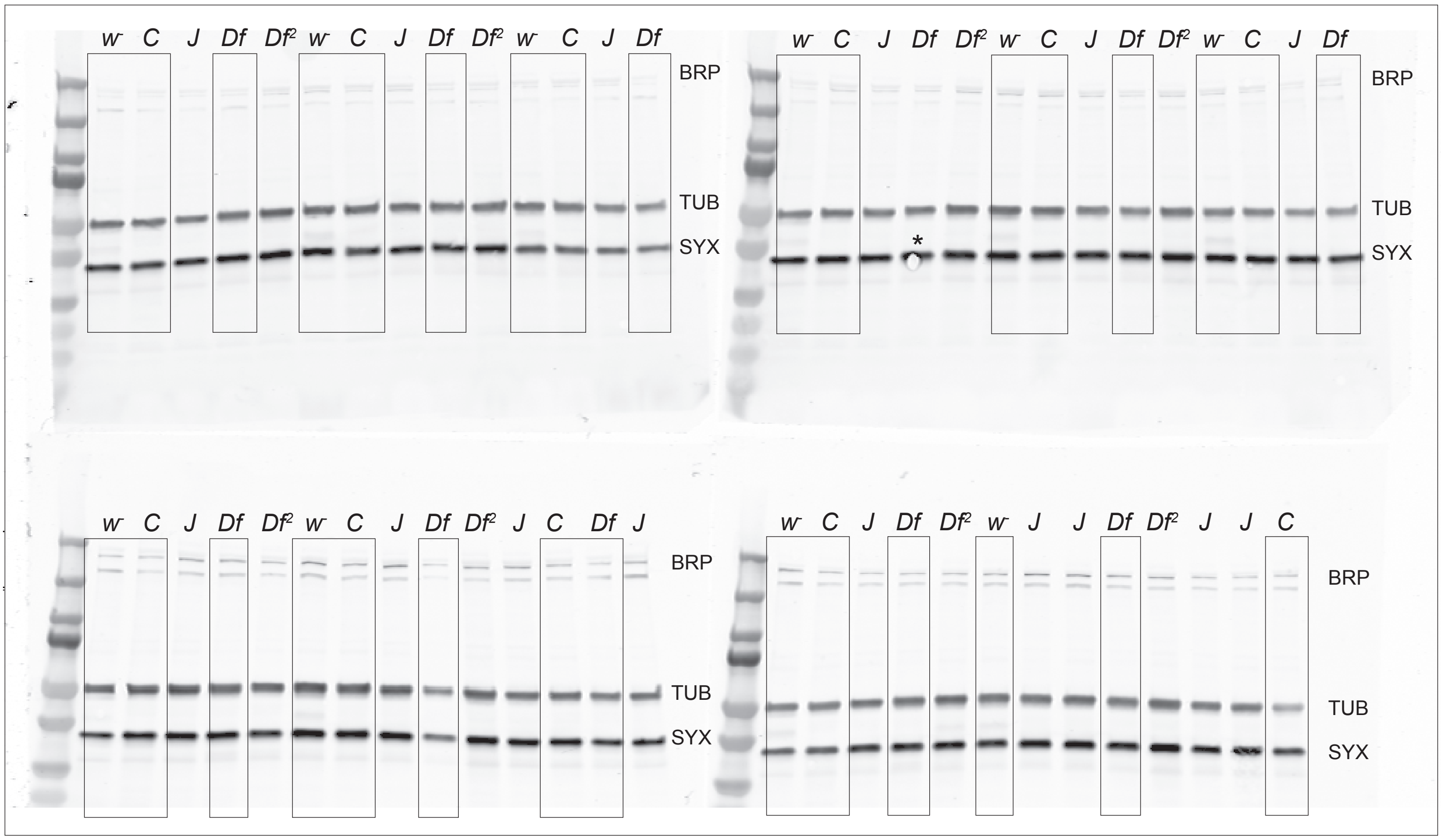

Boxed lanes were analyzed. Letter above lane indicates genotype: w^- (negative control without Cac-GFP), C (Control Cac^{GFP}), J ($Cac^{J/GFP}$ truncation heterozygote, not used in this study) Df ($Cac^{Df/GFP}$ heterozygote, used in this study) Df^2 (A second Cac deficiency heterozygote, not used in this study). All Cac-GFP bands were quantified, and all Syntaxin bands were quantified with the exception of the band marked by an asterisk, in which a bubble prevented accurate quantification of the band. negative controls were used as background subtraction.
