# Supplementary figures and images for: Regulation of presynaptic Ca2+ channel abundance at active zones through a balance of delivery and turnover
Source: eLife. 2022 Jul 14;11:e78648. doi: 10.7554/eLife.78648 (PMC9352347; doi:10.7554/eLife.78648)

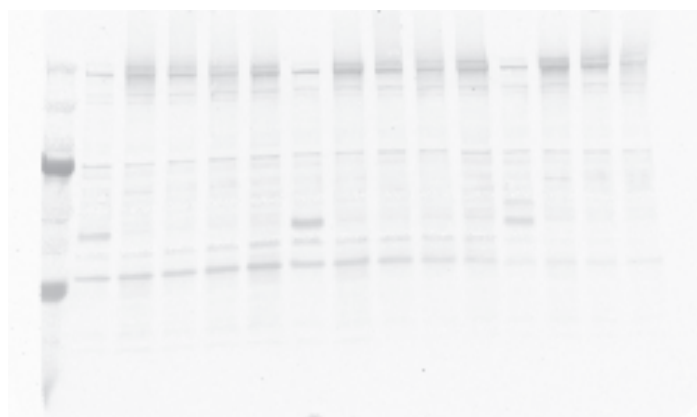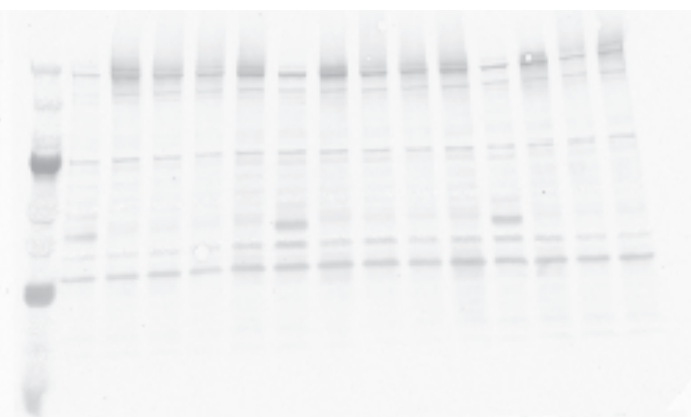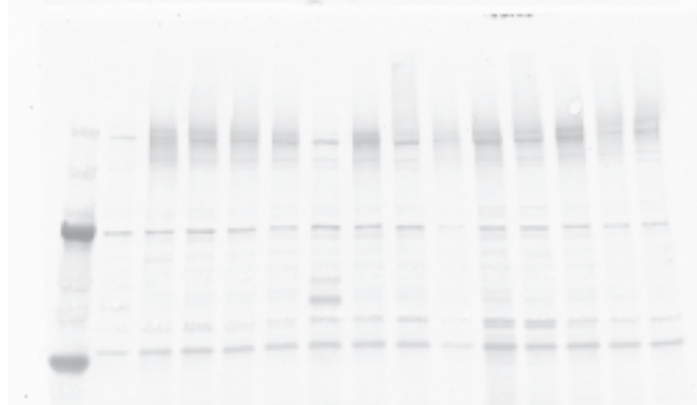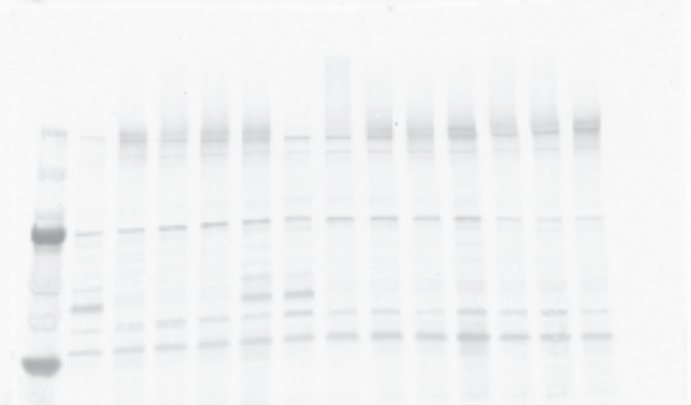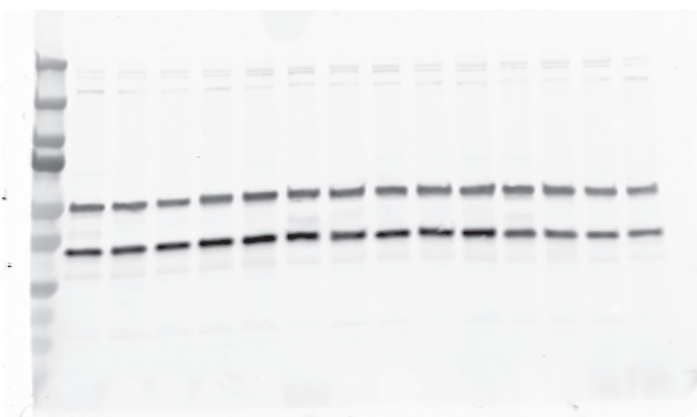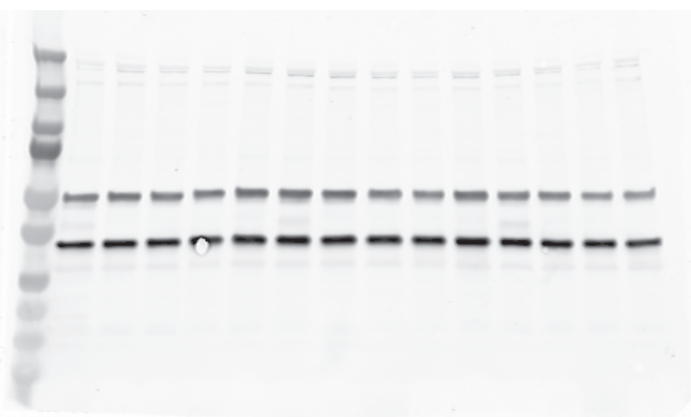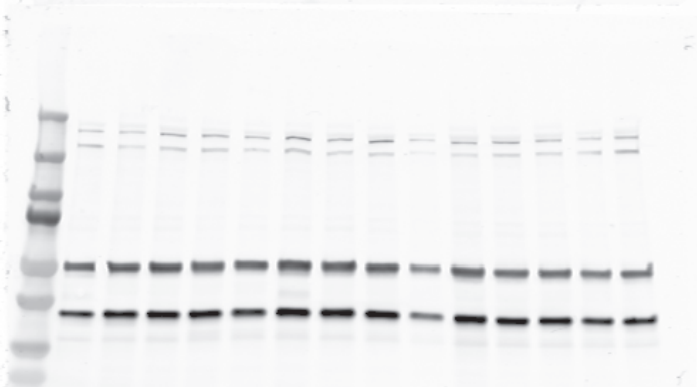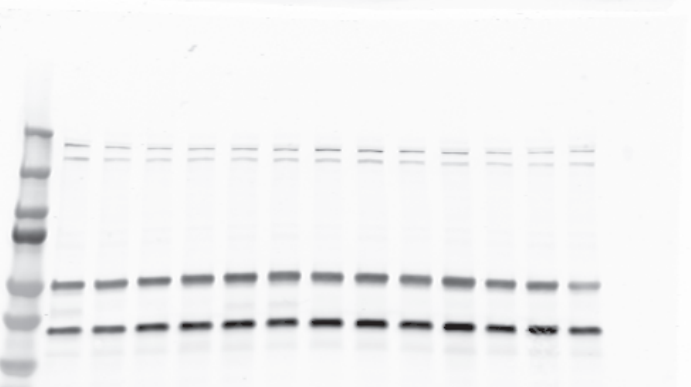

Supplement: Figure 3—source data 3. [file elife-78648-fig3-data3.pdf]

Control

BRP RNAi

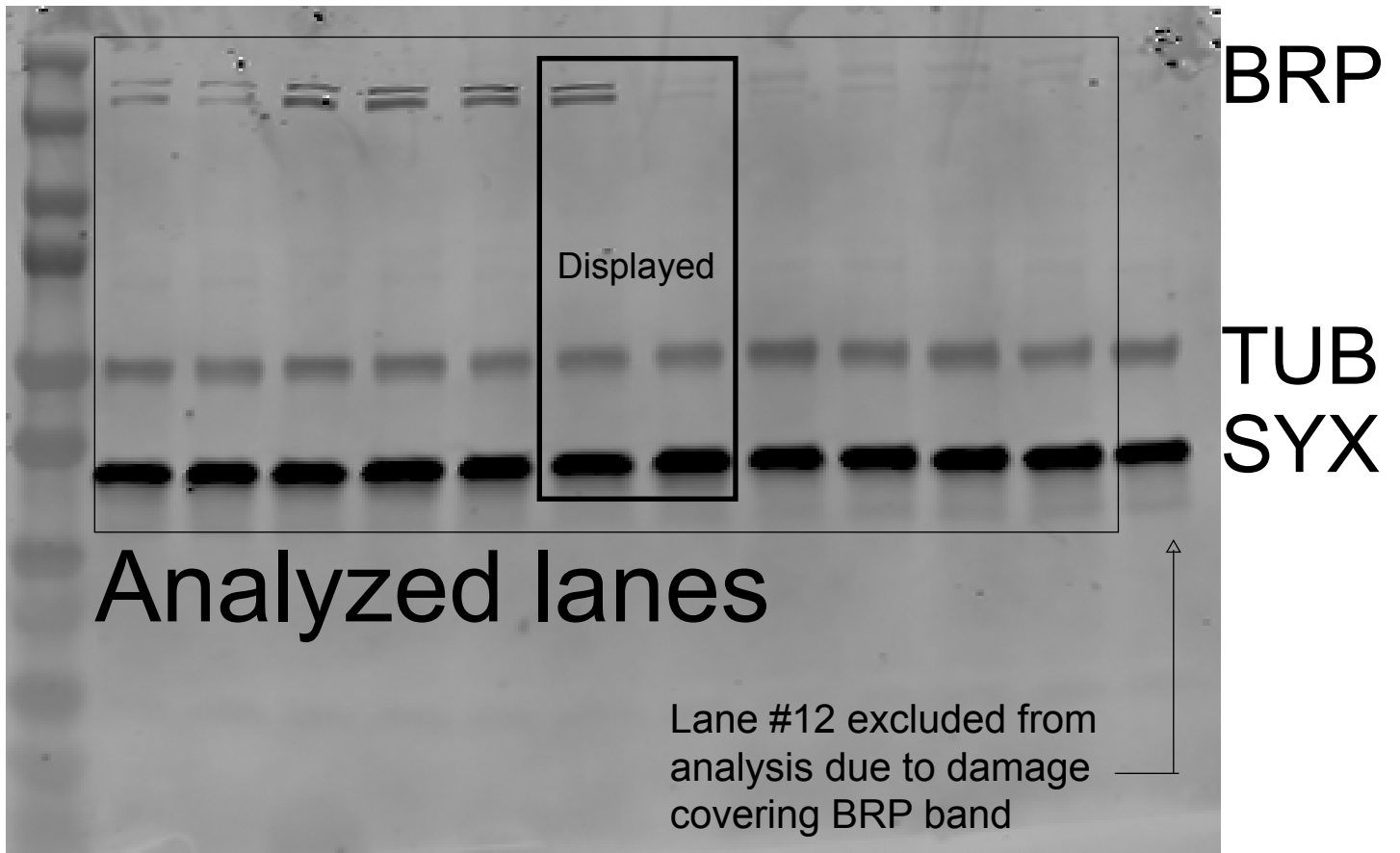

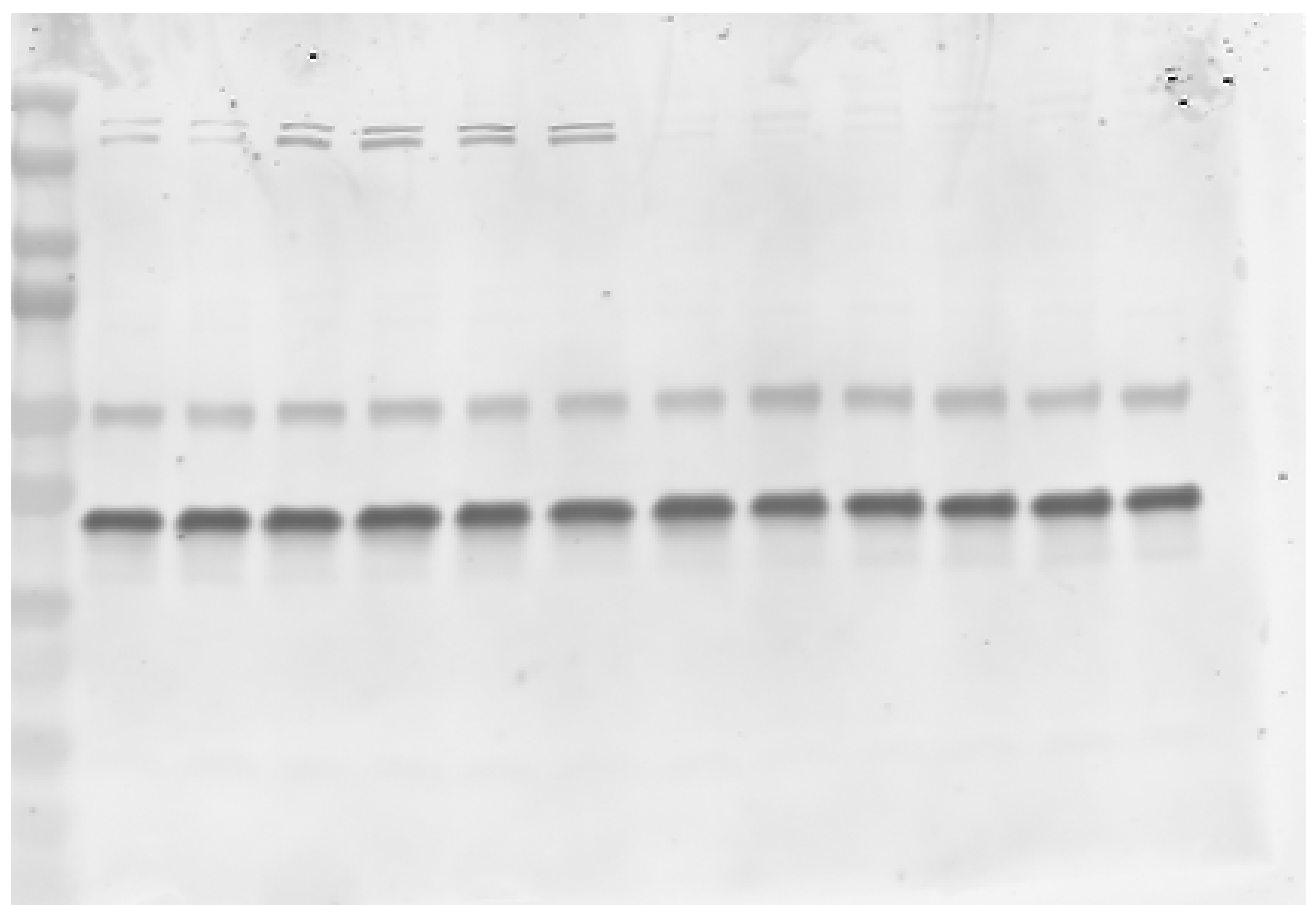

Supplement: Figure 4—source data 3. [file elife-78648-fig4-data3.pdf]
