# Supplementary material for: Regulation of presynaptic Ca2+ channel abundance at active zones through a balance of delivery and turnover
Source: eLife. 2022 Jul 14;11:e78648. doi: 10.7554/eLife.78648 (PMC9352347; doi:10.7554/eLife.78648)

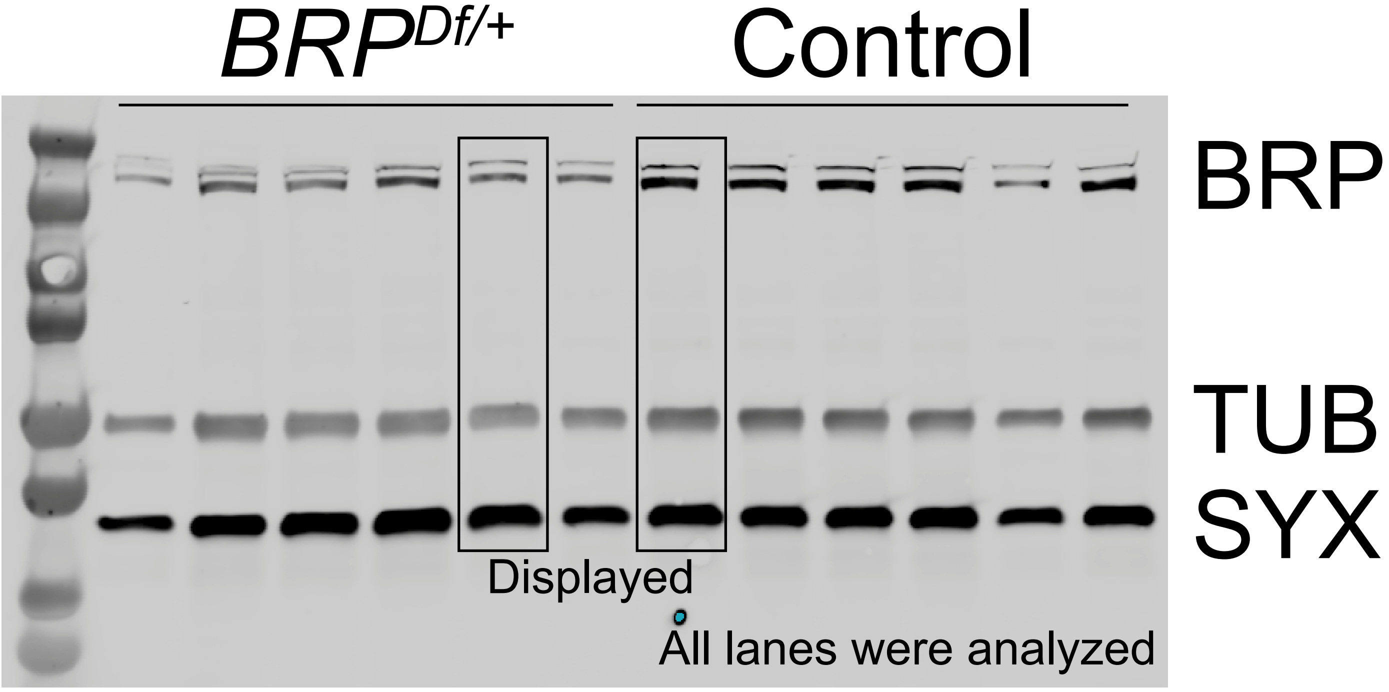

BRP$^{Df/+}$
Control
BRP
TUB
SYX
Displayed
All lanes were analyzed

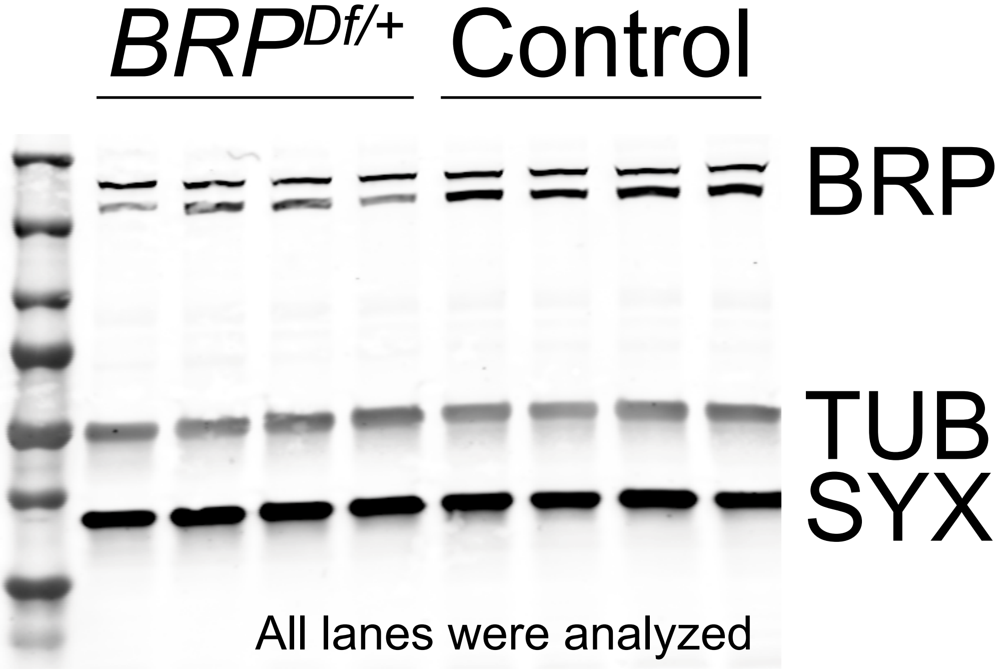

BRP$^{Df/+}$
Control
BRP
TUB
SYX
All lanes were analyzed

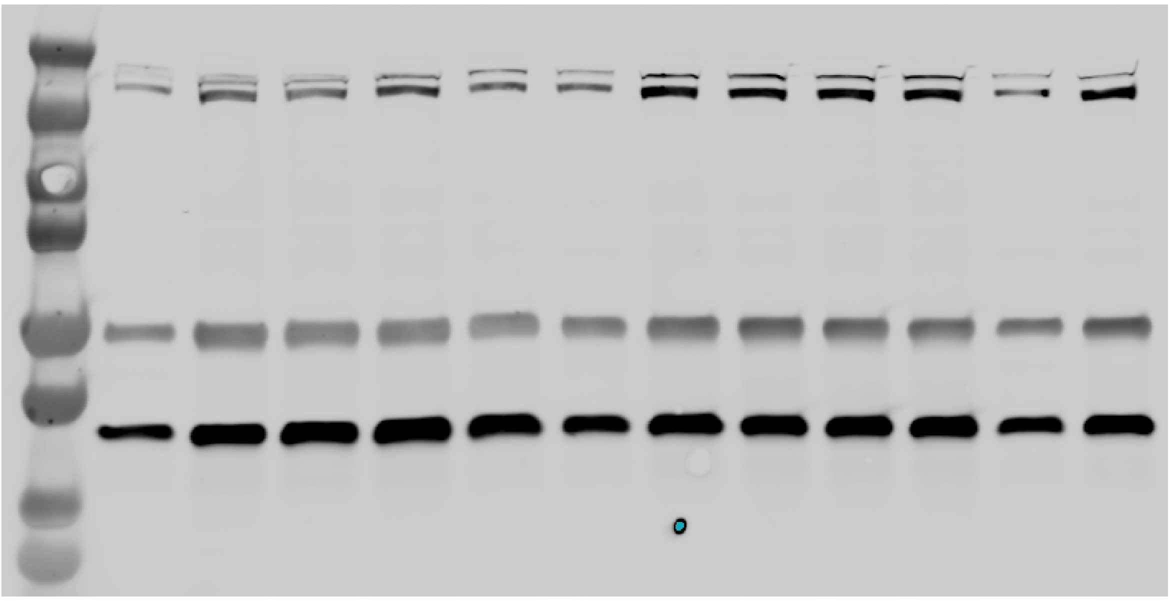

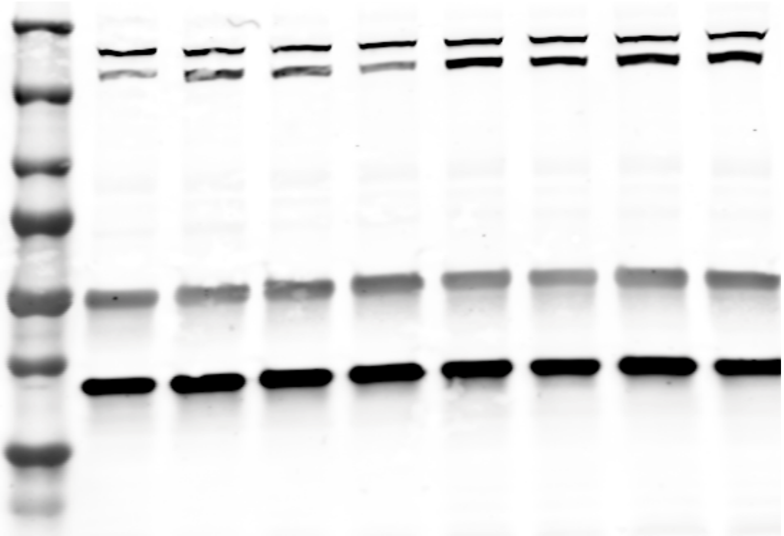

Supplement: Figure 4—source data 2. [file elife-78648-fig4-data2.pdf]
