# Supplementary material for: Regulation of presynaptic Ca2+ channel abundance at active zones through a balance of delivery and turnover
Source: eLife. 2022 Jul 14;11:e78648. doi: 10.7554/eLife.78648 (PMC9352347; doi:10.7554/eLife.78648)
Supplement: Transparent reporting form [file elife-78648-transrepform1.docx]

***eLife’s* transparent reporting form**

We encourage authors to provide detailed information *within their submission* to facilitate the interpretation and replication of experiments. If you have any questions, please contact us: [editorial@elifesciences.org](mailto:editorial@elifesciences.org).

**Sample-size estimation**

- You should state whether an appropriate sample size was computed when the study was being designed
- You should state the statistical method of sample size computation and any required assumptions
- If no explicit power analysis was used, you should describe how you decided what sample (replicate) size (number) to use

Please outline where this information can be found within the submission (e.g., page numbers or figure legends), or explain why this information doesn’t apply to your submission:

Sample-size was not estimated prior to this study, but was chosen based on the standard sample sizes used for each type of experiment in previously published work in the field. Each experiment included a minimum of 5 biological replicates. For imaging and electrophysiology experiments, each biological replicate included data from between 1 and 3 muscle fibers. For Western Blot analysis, each replicate included head extracts from 5 heads. *N* for each experiment is reported in the figure legends and in the Excel spreadsheet “Source Data and Statistics” file.

**Replicates**

- You should report how often each experiment was performed
- You should include a definition of biological versus technical replication
- The data obtained should be provided and sufficient information should be provided to indicate the number of independent biological and/or technical replicates
- If you encountered any outliers, you should describe how these were handled
- Criteria for exclusion/inclusion of data should be clearly stated
- High-throughput sequence data should be uploaded before submission, with a private link for reviewers provided (these are available from both GEO and ArrayExpress)

Please outline where this information can be found within the submission (e.g., page numbers or figure legends), or explain why this information doesn’t apply to your submission:

For imaging and electrophysiology experiments, biological replicates were defined as the number of individual animals tested, and the number of biological replicates is reported in the figure legends. For western blot experiments, biological replicates were defined as the number of samples loaded onto the gel, with each sample prepared from 5 heads. Technical replicates were defined as the number of times the experiment was performed. For Cac-Maple turnover experiments, three technical replicates are described in Figure 7 – figure supplement 1. In one case, an outlier was identified and removed using the default settings of the Identify Outlier function in Prism9. This outlier value is reported in the supplementary data file. For western blots, two lanes were excluded due to damage or a bubble covering the band. These excluded lanes are marked on western blot images in the Excel spreadsheet “Source Data and Statistics” file.

**Statistical reporting**

- Statistical analysis methods should be described and justified
- Raw data should be presented in figures whenever informative to do so (typically when N per group is less than 10)
- For each experiment, you should identify the statistical tests used, exact values of N, definitions of center, methods of multiple test correction, and dispersion and precision measures (e.g., mean, median, SD, SEM, confidence intervals; and, for the major substantive results, a measure of effect size (e.g., Pearson's r, Cohen's d)
- Report exact p-values wherever possible alongside the summary statistics and 95% confidence intervals. These should be reported for all key questions and not only when the p-value is less than 0.05.

Statistical Analysis is described in Materials and Methods and in Figure legends for specific experiments. Raw data are plotted in the figures, with bars corresponding to mean. Sample size (N), mean, SEM, and P values are reported in the Figure Legends. These values, as well as median, quartiles, range, and standard deviation, are reported for each experiment in the Excel spreadsheet “Source Data and Statistics” file.

Please outline where this information can be found within the submission (e.g., page numbers or figure legends), or explain why this information doesn’t apply to your submission:

(For large datasets, or papers with a very large number of statistical tests, you may upload a single table file with tests, Ns, etc., with reference to page numbers in the manuscript.)

**Additional data files (“source data”)**

- We encourage you to upload relevant additional data files, such as numerical data that are represented as a graph in a figure, or as a summary table
- Where provided, these should be in the most useful format, and they can be uploaded as “Source data” files linked to a main figure or table
- Include model definition files including the full list of parameters used
- Include code used for data analysis (e.g., R, MatLab)
- Avoid stating that data files are “available upon request”

Please indicate the figures or tables for which source data files have been provided:

We have included our source data and statistical methods for all figures in the Excel spreadsheet “Source Data and Statistics”.
